# Mono‐planar T‐Hex: Speed and flexibility for high‐resolution 3D imaging

**DOI:** 10.1002/mrm.28979

**Published:** 2021-08-16

**Authors:** Maria Engel, Lars Kasper, Bertram Wilm, Benjamin Dietrich, Franz Patzig, Laetitia Vionnet, Klaas P. Pruessmann

**Affiliations:** ^1^ Institute for Biomedical Engineering ETH Zurich and University of Zurich Zurich Switzerland; ^2^ Translational Neuromodeling Unit, IBT University of Zurich and ETH Zurich Zurich Switzerland

**Keywords:** 3D encoding, algebraic image reconstruction, magnetic field monitoring, spiral imaging

## Abstract

**Purpose:**

The aim of this work is the reconciliation of high spatial and temporal resolution for MRI. For this purpose, a novel sampling strategy for 3D encoding is proposed, which provides flexible k‐space segmentation along with uniform sampling density and benign filtering effects related to signal decay.

**Methods:**

For time‐critical MRI applications such as functional MRI (fMRI), 3D k‐space is usually sampled by stacking together 2D trajectories such as echo planar imaging (EPI) or spiral readouts, where each shot covers one k‐space plane. For very high temporal and medium to low spatial resolution, tilted hexagonal sampling (T‐Hex) was recently proposed, which allows the acquisition of a larger k‐space volume per excitation than can be covered with a planar readout. Here, T‐Hex is described in a modified version where it instead acquires a smaller k‐space volume per shot for use with medium temporal and high spatial resolution.

**Results:**

Mono‐planar T‐Hex sampling provides flexibility in the choice of speed, signal‐to‐noise ratio (SNR), and contrast for rapid MRI acquisitions. For use with a conventional gradient system, it offers the greatest benefit in a regime of high in‐plane resolution <1 mm. The sampling scheme is combined with spirals for high sampling speed as well as with more conventional EPI trajectories.

**Conclusion:**

Mono‐planar T‐Hex sampling combines fast 3D encoding with SNR efficiency and favorable depiction characteristics regarding noise amplification and filtering effects from $T_{2}^{*}$ decay, thereby providing flexibility in the choice of imaging parameters. It is attractive both for high‐resolution time series such as fMRI and for applications that require rapid anatomical imaging.

## 1 INTRODUCTION

High‐resolution volume imaging demands high time efficiency of encoding and acquisition, particularly when temporal resolution is also critical, as in fMRI^1^, ^2^, ^3^ and fQSM,^4^, ^5^ or when contrast preparation is time‐consuming as, for example, in arterial spin labeling (ASL).^6^ For high speed of volume imaging, 3D acquisition is well suited, benefiting from array encoding in three dimensions. One common strategy for fast 3D acquisition is to stack EPI or spiral readouts in 3D k‐space, covering one entire k‐space plane at a time.^7^ This is possible up to a resolution limit, typically somewhat below 1 mm, set by signal decay, intra‐voxel dephasing and gradient performance.

For higher resolution with k‐space stacks of planar readouts, the conventional option is to cover each plane by multiple, interleaved shots.^8^ However, integer increments in the number of interleaves cause abrupt sequence changes that may be disadvantageous. For instance, moving from one to two shots halves the readout time, greatly reducing TR and thus causing leaps in optimal flip angle, contrast, and SNR efficiency. Actual optimization of contrast and SNR yield will generally favor a certain TR regardless of the targeted resolution. Furthermore, due to sequence overhead such as preparation modules, RF excitation, and spoiling, integer steps in the number of interleaves also come with substantial increases in total scan time.

High‐resolution 3D imaging thus calls for covering each k‐space plane with non‐integer numbers of shots larger than one. This problem bears resemblance to the reverse challenge of sampling more than one k‐space plane per shot in the medium‐ to low‐resolution regime. The latter case has recently been tackled by multi‐planar spirals and EPI readouts along tilted hexagonal grids in the indirect dimensions (T‐Hex).^9^

In this work, we propose a modified form of the T‐Hex concept that addresses the high‐resolution scenario, achieving the desired fractional coverage per shot with planar readouts. We demonstrate that mono‐planar T‐Hex increases the flexibility of sequence timing with EPI and spiral acquisition. With spirals, it achieves whole‐brain $T_{2}^{*}$‐weighted imaging with sub‐μl resolution in less than 5 s, using a regular gradient system.

## 2 METHODS

### 2.1 Trajectory design

#### 2.1.1 Tilted hexagonal grids

Fast 3D Fourier encoding is often achieved by stacking identical 2D readouts in the third dimension. EPI and Archimedean spirals then form 2D Cartesian grids in planes orthogonal to the read direction. These planes, which are parallel for EPI and radial about the principal axis of the stack for spirals, shall be referred to as phase‐encoding (PE) planes. However, Cartesian sampling is usuallly suboptimal. Structures with approximately circular or elliptical support are sampled more efficiently on hexagonal grids.^10^, ^11^, ^12^, ^13^ In parallel imaging with undersampling, the greater isotropy of hexagonal patterns improves the conditioning of image reconstruction for a given mean sampling density.^14^, ^15^, ^16^, ^17^

**FIGURE 1:**
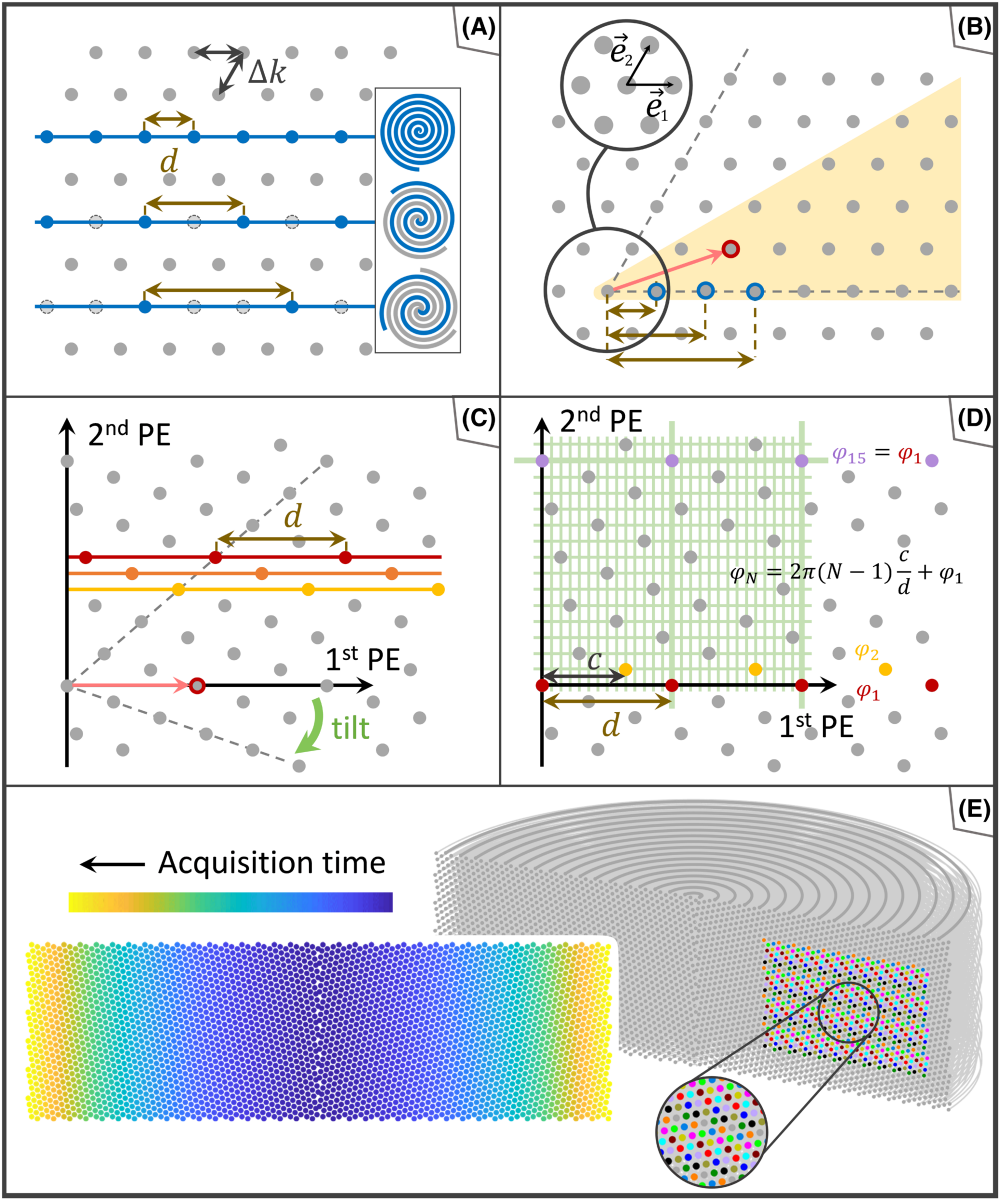
A, Gray dots depict part of the hexagonal grid in a transverse section of a stack of spirals or echo‐planar readouts. Blue dots mark the revolutions acquired within a shot for N = 1, 2, and 3 shots per k‐space plane. B, The hexagonal grid is described by oblique coordinates. All existing distances between grid points are represented in distances of the origin to points in one dodecant (yellow wedge), including its edges. Blue rings mark the distances reflected in (A), and the red ring highlights a distance not available with integer N. C, This distance is used when tilting the grid with respect to the rotational axis of the stack. The FE lines or spiral revolutions acquired in three subsequent shots are highlighted in red, orange, and yellow. D, The hexagonal grid is tilted as shown in (C). Red and yellow dots mark the points of the hexagonal grid that are covered by two subsequent shots. Green lines in the background show that all points of the hexagonal grid lie on nodes of a finer, rectilinear lattice, from which the starting angle of each spiral shot can be derived. Subsequent shots start with linearly progressing angles. The pattern is repetitive. E, Background: An entire mono‐planar T‐Hex stack of spirals cut open. The uppermost shot and the sites of puncture of the spirals are marked in dark gray. The resulting hexagonal grid shows the tilt as visualized in (C) and (D). In a representative patch, grid points that belong to spiral shots with the same phasing are colored the same. Foreground: Cross‐section corresponding to the stack in the upper panel, the acquisition time point is color‐coded, indicating a smooth $T_{2}^{*}$ filter. Owing to the cross‐sectional depiction, “holes” appear on the central k‐space axis. In fact, this region is equally uniformly sampled, since all spirals start from the rotational axis of the cylinder

Spiral and EPI trajectories can readily be stacked in such a way that they cover hexagonal grids in their PE planes (Figure 1A). However, due to the finite feasible length of individual readouts, one shot may not suffice to cover an entire nominal k‐space plane when targeting high resolution. The common solution to this problem is to segment each plane into *N* interleaved shots. However, due to the inflexibility in time allocation arising from *N* being an integer, it is often impossible to optimize the sequence timing for contrast and SNR as discussed more closely in the Supporting Information.

Towards greater flexibility of segmentation, a key parameter is the PE increment *d* of each individual shot, which is the spacing of readout lines in EPI and the radial increment per turn for a spiral readout. For conventional segmentation, *d* is constrained to integer multiples of the lattice constant *Δk* as shown in Figure 1A. However, other distances occur on the hexagonal grid as illustrated in Figure 1B. Defining the hexagonal grid by the basis vectors ${\vec{e}}_{1}$ and ${\vec{e}}_{2}$, a greater variety of vector lengths results from forming integer combinations of ${\vec{e}}_{1}$ and ${\vec{e}}_{2}$:

(1) $$\vec{v}=M_{1}\cdot {\vec{e}}_{1}+M_{2}\cdot {\vec{e}}_{2}$$

Due to the symmetries of the grid, all possible distances are represented by vectors from the origin to points in the first dodecant marked yellow in Figure 1B (*M*_1_ ≥ *M*_2_ ≥ 0).

This greater choice of distances can be exploited to increase the flexibility of segmentation by selecting a grid point with favorable distance *d* from the origin (eg, *M*_1_ = 2, *M*_2_ = 1, marked red in Figure 1B) and tilting the grid such that the corresponding vector $\vec{v}$ is orthogonal to the principal axis of the stack (2nd PE axis in Figure 1C). For all choices of $\vec{v}$, the points on the tilted hexagonal grid fall onto a finer, rectilinear grid (green lines in Figure 1D), as derived in the Appendix of Ref. [9].

For optimal imaging performance, the eventual sampling density must account for the required FOV and the parallel imaging capability of the array used. Either or both may be anisotropic, calling for different densities in the two PE directions. To implement these, the tilted hexagonal pattern is rescaled in the two dimensions and cropped according to the desired resolution.

#### 2.1.2 Application to spirals and EPI

The Archimedean spiral underlying a stack of spirals (Figure 1E) needs to be designed only once. It is then rotated and shifted along the 2nd PE direction according to the respective T‐Hex pattern, involving fixed shot‐to‐shot phase and shift increments. Like in the case of multi‐planar T‐Hex readouts,^9^ identical radial progression of all interleaves ensures smooth $T_{2}^{*}$ weighting. Gained flexibility of the increment *d* in the 1st PE direction corresponds to flexibility in slab thickness in the multi‐planar case. Similarly, the repetitiveness of blipping in the multi‐planar case corresponds to repetition of the sequence of phase offsets in the mono‐planar scenario. The number of shots within this sequence reads

$$\frac{2\left(M_{1}^{2}+M_{2}^{2}+M_{1}M_{2}\right)}{\text{gcd}\left(2M_{2}+M_{1},\;2M_{1}+M_{2}\right)}$$

In the example shown in Figure 1D, for instance, the sequence has length 14. Blipping as such and any related deceleration are not necessary in the mono‐planar case.

All of these considerations apply equally to the EPI variant, with shot‐to‐shot in‐plane shifts instead of rotations as well as corresponding echo time shifts for smooth $T_{2}^{*}$ weighting. The echo time shift is proportional to the ratio of the in‐plane shift (*c* in Figure 1D) and the spacing *d* and scales with the temporal spacing of readout lines, analogous to conventional segmented EPI.^18^

Based on the resulting k‐space trajectories, the gradient waveforms were computed so as to minimize their duration within gradient‐strength and slew‐rate constraints.^19^

### 2.2 Speed and flexibility

To assess the utility of the proposed approach, resulting scan times and underlying acquisition times per shot, *T_AQ_*, were calculated for a typical whole‐brain imaging scenario with FOV = 24 × 24 × 12 cm^3^ and varying in‐plane resolution of 0.4 mm, 0.6 mm, and 0.8 mm, and 2 mm through‐plane resolution. Stacks of mono‐planar T‐Hex spirals were generated as described above, varying the tilt according to all points in the yellow wedge in Figure 1B with *d* up to 7.6 Δ*k*, which corresponds to *M*_1_ = 7, *M*_2_ = 1. For comparison, spirals were also stacked conventionally, forming Cartesian grids in PE planes. Hexagonal and Cartesian grids were chosen such as to encode the same ellipse in the image domain with net undersampling by a factor of R = 8. An overhead of 13 ms per shot was included to account for typical excitation, fat suppression, and spoiling modules.

### 2.3 In‐vivo experiments

Experiments were carried out on a 7T whole‐body MRI system (Achieva, Philips Healthcare, Best, The Netherlands), using a quadrature‐transmit and 32‐channel receive head setup (Nova Medical, Wilmington, USA). The system was operated in a mode offering gradient amplitude up to 31 mT/m and a maximum slew‐rate of 200 T/m/s.

Mono‐planar spiral and EPI T‐Hex readouts were included in a common gradient echo sequence (parameters shown in Table 1, *M*_1_ = 2, *M*_2_ = 1). Additional 3D multi‐echo spin‐warp scans served for B_0_ and coil sensitivity mapping. T‐Hex EPI included a SPIR fat suppression module.^20^ K‐space trajectories were pre‐recorded using a field camera (Skope Magnetic Resonance Technology, Zurich, Switzerland) made from 16 ^1^H NMR field probes (*T_1_* = 62 ms,$T_{2}^{*}$ = 36 ms),^21^, ^22^, ^23^, ^24^ operated with the console described in Ref. [25].

**TABLE 1:** Sequence parameters

Trajectory type	FOV [cm^3^]	R	Resolution	TR [ms]	TE [ms]	No. of shots	Total scan duration
T‐Hex spiral‐out	24 × 24 × 12	8	0.6 × 0.6 × 2 mm^3^	56	15	86	4.8 s
T‐Hex spiral‐out	24 × 24 × 12	8	0.6 × 0.6 × 2 mm^3^	66	25	86	5.7 s
T‐Hex EPI	24 × 24 × 12	8	0.7 × 0.7 × 2 mm^3^	50	20	86	4.3 s
Cartesian spin‐warp	24 × 24 × 13.2	1	1.5 mm^3^	31	1.95‐6.80	10500	5 min 26 s

Data were collected from two healthy volunteers according to the applicable ethics regulation. Image reconstruction was performed using a recent 3D extension^9^ of algebraic image reconstruction based on an expanded signal model.^26^, ^27^ In reconstructed images, residual weighting by net array sensitivity was removed by bias field correction (SPM12, http://www.fil.ion.ucl.ac.uk/spm/software/spm12/).^28^ Background phase was removed using the STI Suite toolbox (https://people.eecs.berkeley.edu/~chunlei.liu/software.html), based on the V‐SHARP algorithm.^29^

## 3 RESULTS

**FIGURE 2:**
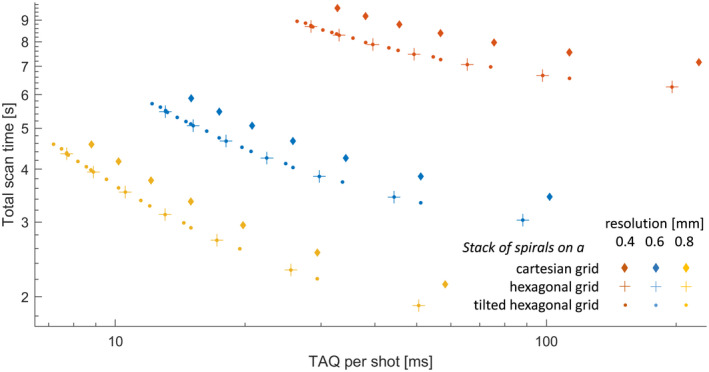
For three in‐plane resolutions, the acquisition time for different stacks of spirals was computed and plotted against their total scan time. The latter comprises an overhead of 13 ms per shot, accounting for typical slab excitation, fat suppression and spoiling modules. For the trajectory design, a FOV of 24 × 24 × 12 cm^3^ and R = 8 were assumed

Figure 2 shows the results of the study described in Section 2.2, plotting total scan time against the underlying *T_AQ_* per shot. First, this diagram illustrates the expected speed benefit of hexagonal sampling over conventional stacks in all resolution regimes. More importantly, it also confirms the benefit of tilting the hexagonal grids, which enhances the flexibility of how much k‐space volume to cover per shot.

**FIGURE 3:**
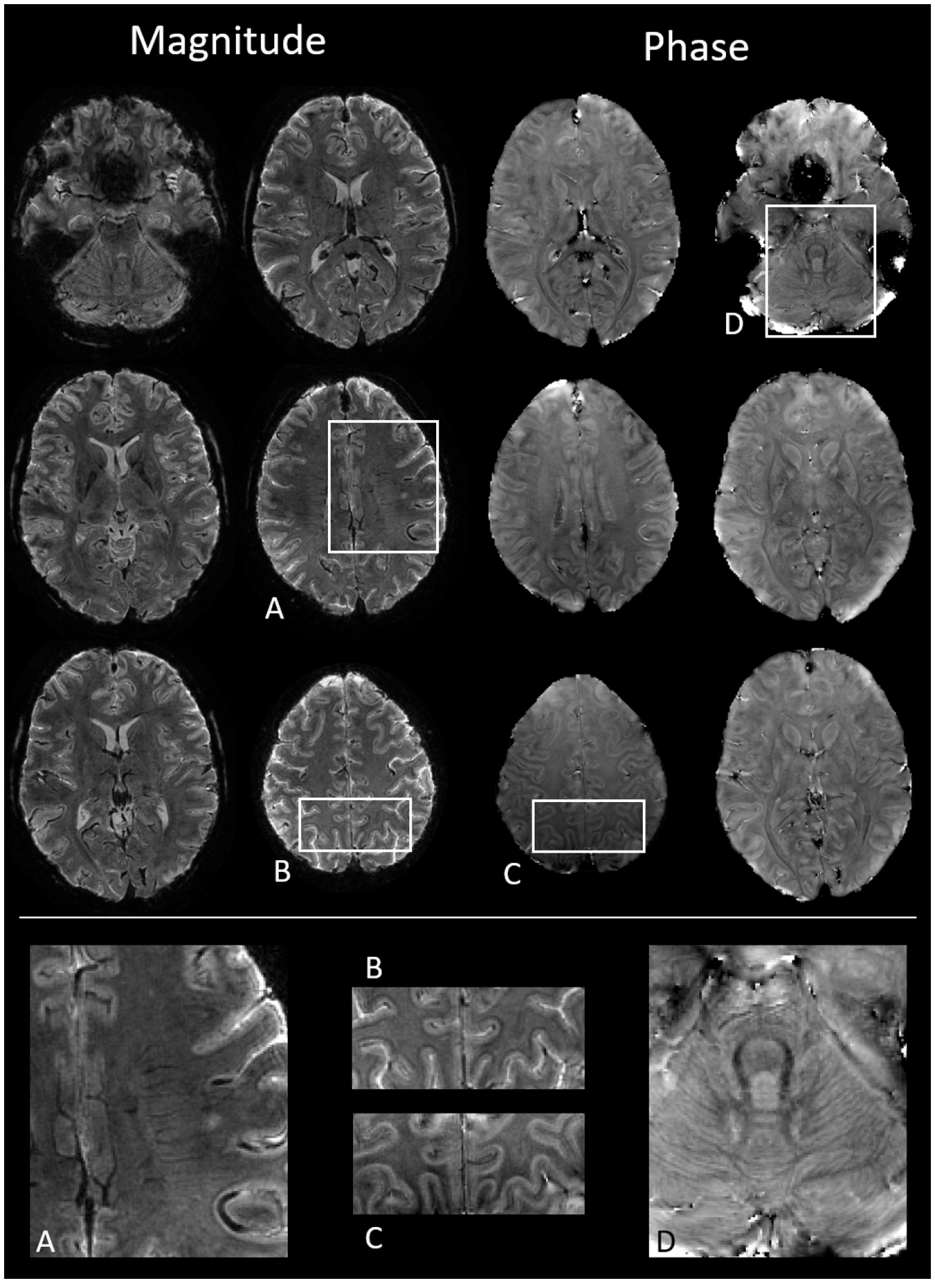
Slices from in‐vivo data acquired with mono‐planar T‐Hex spiral‐out. The whole brain is covered with 0.6 × 0.6 × 2 mm^3^ resolution in 5.7 s, TE = 25 ms. The upper part shows magnitude images of six selected slices on the left, with corresponding phase images shown on the right. In the lower part, enlarged details are displayed

**FIGURE 4:**
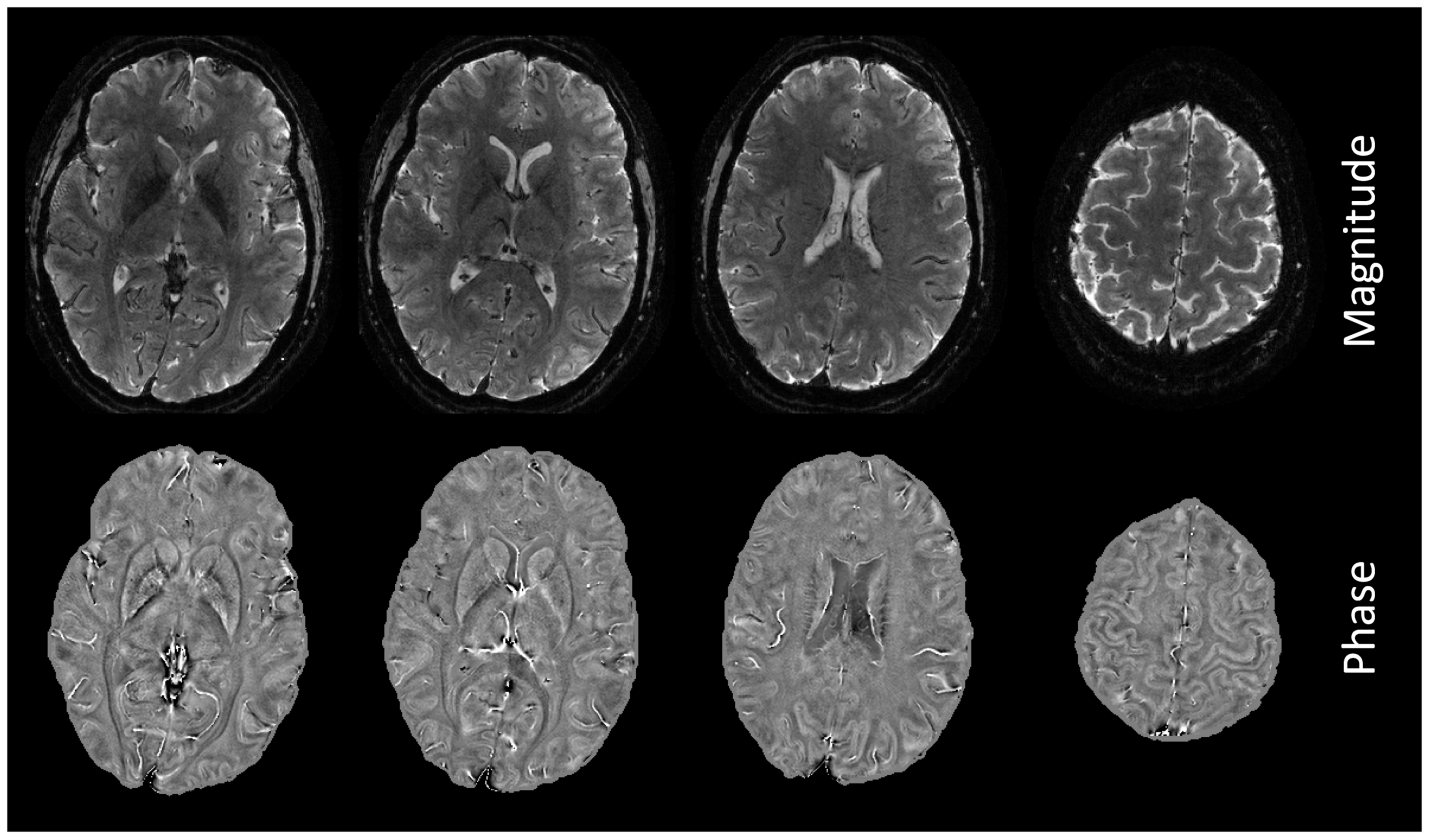
4 Mono‐planar T‐Hex EPI with 0.7 × 0.7 × 2 mm^3^ nominal resolution and TE = 20 ms, acquired in 4.3 s

Supporting Information Figure S2 shows selected transverse slices from the first mono‐planar T‐Hex spiral‐out scan, resolving 720‐nanoliter voxels in 4.8 s. Data obtained with the same trajectories but stronger $T_{2}^{*}$ weighting and sensitivity to susceptibility differences (TE = 25 ms) are shown in Figure 3, including image phase and enlarged details. Figure 4 presents slices from the mono‐planar T‐Hex EPI scan, achieving somewhat lower resolution, at 1 microliter per voxel, in only 4.3 s.

## 4 DISCUSSION

Planar readouts along tilted hexagonal grids permit dividing the volume of a nominal k‐space plane into fractions per shot other than inverse integers, while maintaining uniform sampling density. The key benefit of this approach is greater flexibility of optimizing the duration of each readout in 3D imaging. Such flexibility is instrumental not only for minimizing overall scan duration for given signal lifetime but also for optimizing contrast and SNR efficiency. Facilitating the use of multiple shots per nominal plane, the proposed strategy applies to the case of 3D imaging with relatively high resolution. The gain in flexibility is greatest in the regime of few shots per nominal plane where integer steps in the number of shots are the largest in relative terms.

Mono‐planar T‐Hex shares key properties with the kindred multi‐planar T‐Hex scheme, which enables fractional coverage of more than one nominal plane per shot for the case of moderate spatial resolution.^9^ Like the latter, mono‐planar T‐Hex spirals and EPI reconcile flexibility of segmentation with high average k‐space speed, uniform density, and smooth $T_{2}^{*}$ weighting for time‐efficient acquisition with near‐optimal conditioning of reconstruction and tolerance to signal decay over long readouts. Unlike their multi‐planar counterparts, the mono‐planar readouts do not require blips to switch planes and potential slow‐down to reduce associated sampling gaps.^9^

Demonstrated here with regular spiral‐out and EPI trajectories, the proposed strategy is equally applicable to variations of these readouts such as inward spirals^30^ and variable‐density spirals^31^ and EPI.^32^ It is also expected to apply to multi‐band imaging whose encoding requirements are essentially the same as for 3D imaging.^33^

As illustrated in Figure 1D, the T‐Hex sampling pattern repeats in the direction of the stack so that the sequence of readout trajectories is equally repetitive except for the prephaser gradient. As with regular stacks, this property may facilitate trajectory calibration, either by reducing the number of pre‐measurements required or, in the case of concurrent trajectory recording with NMR probes, by reducing the rate of probe re‐excitation in favor of T_1_ recovery.^27^ As also shown in Figure 1D, all tilting scenarios lead to hexagonal grids that each fall onto some finer Cartesian grid, which is not tilted with respect to the stack's principal axis. With EPI readouts, this property could be of utility for image reconstruction, permitting direct use of FFT without gridding, albeit with a FOV dictated by the embedding Cartesian grid. When this FOV is too small, it could be increased by refining the k‐space grid by integer factors. When it is too large, the excess size could offset the speed benefit of mere FFT. Alternatively, the primary hexagonal grid could be exploited by hexagonal fast Fourier transform (FFT) for image reconstruction, leading to hexagonal image representation.^11,34^ Either approach will require that the trajectories are played out highly accurately.

Modifications of fast 3D acquisition by relative rotation of spiral arms or shifting of echo‐planar readouts have been explored in a number of previous contributions. Refs. [35, 36] describe the heuristic selection of subsets of points on a regular Cartesian grid for favorable packing of neighboring interleaves, achieving approximate uniformity for specific degrees of segmentation. Approximate uniformity can also be achieved with spiral readouts with linear, heuristically chosen, or golden‐angle increments of rotation as k_z_ increases.^37^, ^38^ The T‐Hex approach differs from these strategies in that it defines grids of uniform density for variable segmentation. This relationship is connatural to that between schemes for covering multiple k‐space planes per shot described in Refs. [39, 40, 41] and the multi‐planar T‐Hex approach.^9^ Approximately hexagonal or otherwise taylored patterns have also been explored for sampling domains other than common k‐space such as k‐t space for dynamic imaging,^42^ multi‐contrast imaging,^43^ or T_2_ shuffling,^44^ as well as k‐k_v_‐t space for velocity spectroscopy^45^ and k‐k_PSF_ space for off‐resonance correction.^46^

For time efficiency, the proposed approach seeks to combine high average k‐space speed with uniform sampling density for maximal acceleration within g‐factor limits. Another strategy of containing g‐factors is to start from strongly undersampling baseline trajectories and include oscillatory lateral excursions to reduce sampling gaps as, for example, in bunched phase encoding,^47^ zig‐zag sampling,^48^ EPI or spirals with sinusoidal perturbation,^49^, ^50^ and wave‐CAIPI.^51^, ^52^ However, such oscillation is limited by the feasible speed of gradient switching. For maximal gradient strength, *G*_max_, and slew rate, *S*_max_, the smallest radius of curvature that a trajectory can have at full speed is $\gamma G_{\max}^{2}/S_{\max}$, which is typically in the same order of magnitude as the entire k‐space range to be sampled. As a consequence, for oscillatory perturbations to accomplish substantial additional encoding, the underlying scan must exhibit relatively low k‐space speed in the first place,^53^ at the expense of overall scan time. For instance, whole‐brain wave‐CAIPI acquisition with resolution of 1 × 1 × 2 mm^3^ and 9‐fold acceleration has been reported to take 40 s,^51^ compared to 4.3 s with T‐Hex EPI as reported here, achieving higher resolution (0.7 × 0.7 × 2 mm^3^) in the same FOV at eight‐fold acceleration, with readouts of 33 ms vs. 14 ms. Orthogonal gradient oscillation can add a small amount of encoding capability also to amplitude‐limited EPI and spirals, reflecting slight contributions to net k‐space speed. However, such gain is ambivalent in that it relies on the fact that physically separate gradient coils and amplifiers are subject to individual current and voltage limitations. With angulation, orthogonal oscillating gradients will not yield the same benefit.

The benefits of mono‐planar T‐Hex acquisition in terms of scan speed, SNR yield, and contrast are greatest for 3D imaging at relatively high resolution. The bare speed advantage makes it particularly suitable for temporally resolved imaging like BOLD fMRI, ASL fMRI, and fQSM. With an emphasis rather on spatial resolution, it holds promise for fast structural gradient echo scans with plain $T_{2}^{*}$ weighting, for SWI, and for QSM. The speed benefit is greatest for sequences with significant overhead for signal preparation such as MPRAGE and ASL, which readily lend themselves to 3D EPI and spiral readouts. In addition, more efficient 3D encoding is attractive also for spin‐echo imaging. Based on EPI and spiral readouts, GRASE (gradient and spin echo)^54^, ^55^, ^56^ is a natural area of application, with utility particularly for FLAIR (fluid‐attenuated inversion recovery)^57^ and ASL.^58^, ^59^ However, mono‐planar T‐Hex should also lend itself to plain Cartesian 3D RARE (rapid acquisition with relaxation) imaging,^60^, ^61^ exploiting even time progression in k‐space for smooth T_2_ weighting. Another spin‐echo case of particular interest is diffusion‐weighted imaging, which could benefit significantly due to its demand for spatial resolution and substantial overhead for diffusion weighting.^62^ Insofar as inter‐shot phase inconsistency due to microscopic motion during diffusion weighting can be managed, for example, by navigation,^63^, ^64^ array acquisition^65^ or compensation,^66^ diffusion imaging is amenable to 3D acquisition and T‐Hex sampling. For all potential applications, high B_0_ strengthens the case by boosting baseline sensitivity in favor of targeting high resolution at high speed.

## CONFLICT OF INTEREST

Klaas Pruessmann holds a research agreement with and receives research support from Philips. He is a shareholder of Gyrotools LLC. Bertram Wilm and Benjamin Dietrich are employees of Skope Magnetic Resonance Technologies AG.

## Supporting information


**FIGURE S1** SNR in dependence of $T_{2}^{*}$ and acquisition time per shot. The orange line marks those acquisition times which maximize the SNR for a given $T_{2}^{*}$
**FIGURE S2** Images acquired with T‐Hex spiral‐out. The whole brain is covered with 0.6 × 0.6 × 2 mm^3^ resolution in 4.8 s, TE = 15 ms. Red lines mark the position of the displayed slices
