# Supplementary material for: Mono‐planar T‐Hex: Speed and flexibility for high‐resolution 3D imaging
Source: Magn Reson Med. 2021 Aug 16;87(1):272–80. doi: 10.1002/mrm.28979 (PMC9292510; doi:10.1002/mrm.28979)
Supplement: Supplementary file 1 — FIGURE S1 SNR in dependence of T2∗ and acquisition time per shot. The orange line marks those acquisition times which maximize the SNR for a given T2∗ FIGURE S2 Images acquired with T‐Hex spiral‐out. The whole brain is covered with 0.6 × 0.6 × 2 mm3 resolution in 4.8 s, TE = 15 ms. Red lines mark the position of the displayed slices [file MRM-87-272-s001.pdf]

1 SNR efficiency

The need for flexibility regarding readout strategies shall be demonstrated exemplarily with a simple calculation of SNR efficiency for a completely spoiled gradient-recalled-echo (GRE) sequence. We assume that multiple interleaves cover k-space in such a way that the sampling density is largely uniform. Similar to previous studies on this matter (1–4), the goal is to find the acquisition length of each interleave shot, T_{AQ}, that optimizes SNR for a given T_2^*. This is done disregarding concomitant effects of the acquisition length on actual resolution and artifact level for the moment.

Let us consider a completely spoiled gradient echo sequence, operating in the regime of $TR \ll T_1$, which is commonly the case in 3D in-vivo imaging. According to Ref. (5), the steady-state magnetization using the Ernst angle is then governed by

$$M_{SS} \propto \sqrt{TR}. \quad [1]$$

For simplicity, we neglect overheads such as excitation or spoiling modules. Thus all scan time is used for signal acquisition and

$$TR = T_{AQ}. \quad [2]$$

If nominal resolution, FOV, undersampling factor, and total scan time are kept fixed, only the number of shots, N, among which the k-space volume in question is distributed, is variable, and for their length, it follows

$$T_{AQ} \propto \frac{1}{N}. \quad [3]$$

Hence, we can assume that total acquisition time and thus bandwidth remain fixed, and the SNR varies only with the signal and not with the noise:

$$SNR \propto M_{SS} \cdot D \quad [4]$$

D accounts for signal decay during acquisition. As the latter is only partly included in the common approach of considering exponential decay until the echo time, we use here, instead, the average decay over an extended readout

$$D = \frac{1}{T_{AQ}} \int_0^{T_{AQ}} e^{-t/T_2^*} \, dt. \quad [5]$$

This assumes that all readouts start at the same time, in agreement with Eq. 2. Working out Eq. 5 and substituting it together with Eq. 1 into Eq. 4 results in

$$SNR \propto \frac{T_2^*}{\sqrt{T_{AQ}}}\left(1 - e^{-\frac{T_{AQ}}{T_2^*}}\right), \qquad [6]$$

which reaches its maximum at

$$T_{AQ} = -\left(W_{-1}\left(\frac{1}{2\sqrt{e}}\right) + 1/2\right) T_2^* \approx 1.26\, T_2^*, \qquad [7]$$

with the Lambert W function. Figure S1 shows the SNR relation of Eq. 6, where the orange curve indicates the SNR-maximizing values of T_{AQ}, depending linearly on T_2^* as described in Eq. 7.

The provided calculation indicates that for optimal SNR, readout lengths should be on the order of T_2^*, and that much shorter readouts are particularly inefficient in this regard. Note that in this simplified approach, the assumption of negligible sequence overhead from excitation and spoiling does not hold true for relatively short readouts, which would need a more involved calculation.

Furthermore, in a real scanning situation, sequence optimization would comprise effective resolution, contrast, and artifact proneness, in addition to SNR.

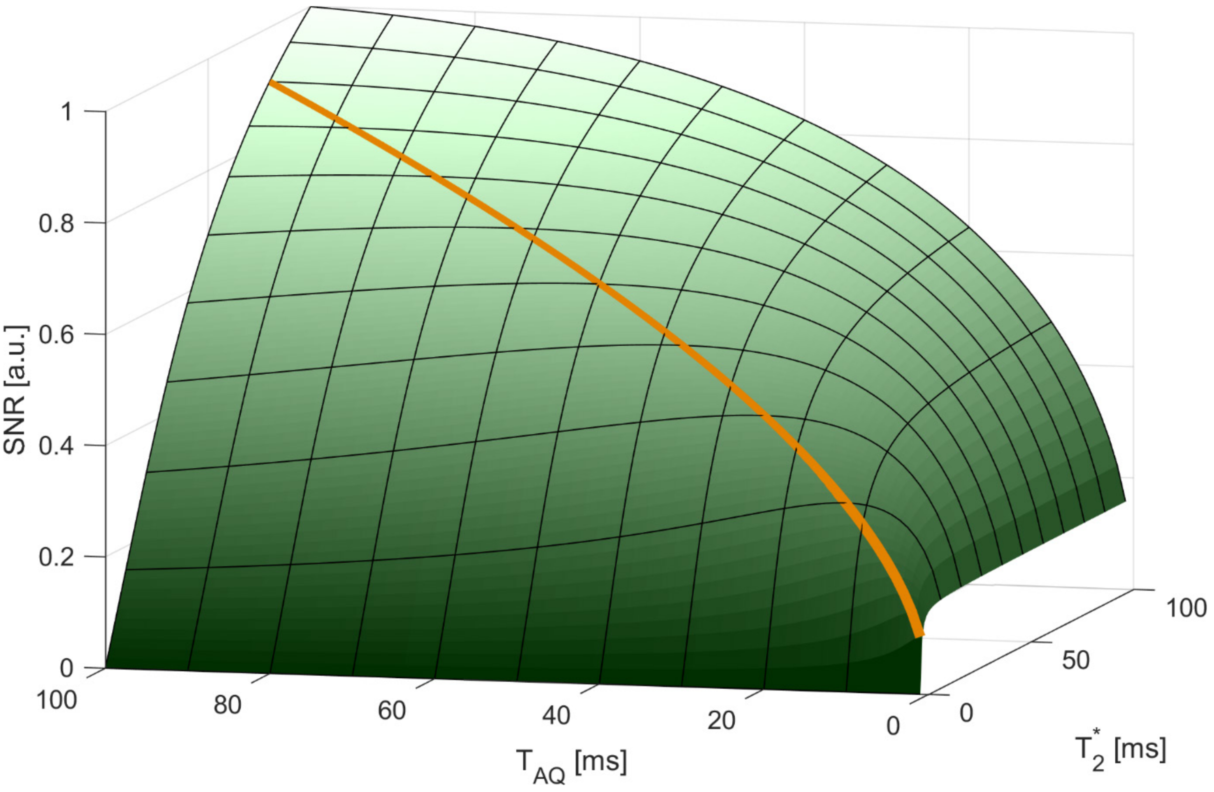

***Figure S1**: SNR in dependence of T_2* and acquisition time per shot. The orange line marks those acquisition times which maximize the SNR for a given T_2*.*

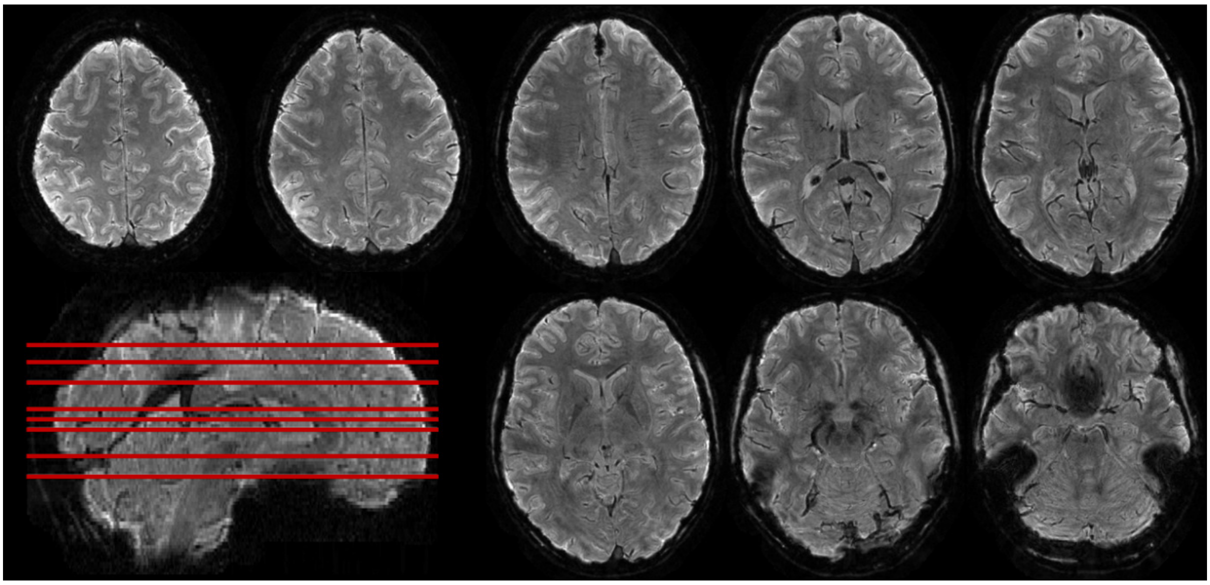

Figure S2: *Images acquired with T-Hex spiral-out. The whole brain is covered with 0.6 x 0.6 x 2 mm^3 resolution in 4.8 s, TE = 15 ms. Red lines mark the position of the displayed slices.*

2 References

1. Callaghan PT, Eccles CD. Sensitivity and resolution in NMR imaging. Journal of Magnetic Resonance (1969) 1987;71:426–445 doi: 10.1016/0022-2364(87)90243-5.

2. Rahmer J, Börnert P, Groen J, Bos C. Three-dimensional radial ultrashort echo-time imaging with T2 adapted sampling. Magnetic Resonance in Medicine 2006;55:1075–1082 doi: 10.1002/mrm.20868.

3. Qin Q. Point spread functions of the T2 decay in k-space trajectories with long echo train. Magnetic Resonance Imaging 2012;30:1134–1142 doi: 10.1016/j.mri.2012.04.017.

4. Froidevaux R, Weiger M, Rösler MB, et al. High-resolution short-T2 MRI using a high-performance gradient. Magnetic Resonance in Medicine n/a doi: 10.1002/mrm.28254.

5. Weiger M, Pruessmann KP. Short-T2 MRI: Principles and recent advances. Progress in Nuclear Magnetic Resonance Spectroscopy 2019;114–115:237–270 doi: 10.1016/j.pnmrs.2019.07.001.
